# Authors’ Response to Peer Reviews of “Thyroid Hyperplasia and Neoplasm Adverse Events Associated With Glucagon-Like Peptide-1 Receptor Agonists in the Food and Drug Administration Adverse Event Reporting System: Retrospective Analysis”

**DOI:** 10.2196/58273

**Published:** 2024-05-01

**Authors:** Tigran Makunts, Haroutyun Joulfayan, Ruben Abagyan

**Affiliations:** 1 Skaggs School of Pharmacy and Pharmaceutical Sciences, University of California San Diego, La Jolla, CA, United States; 2 University of California San Diego, La Jolla, CA, United States

**Keywords:** monotherapy, GLP, FDA, adverse, reporting, thyroid, cancer, oncology, cancers, neoplasm, neoplasms, drug, drugs, pharmacy, pharmacies, pharmacology, pharmacotherapy, pharmaceutic, pharmaceutics, pharmaceuticals, pharmaceutical, medication, medications, glucagon-like peptide-1, Food and Drug Administration

*This is the authors’ response to peer-review reports for “Thyroid Hyperplasia and Neoplasm Adverse Events Associated With Glucagon-Like Peptide-1 Receptor Agonists in the Food and Drug Administration Adverse Event Reporting System: Retrospective Analysis.”*

## Round 1 Review

### Anonymous [1]

#### General Comments

*In this manuscript titled “Thyroid Hyperplasia and Neoplasm Adverse Events Associated With Glucagon-Like Peptide-1 Receptor Agonists in the Food and Drug Administration Adverse Event Reporting System: Retrospective Analysis” [2], the authors analyzed over 18 million reports from the Food and Drug Administration (FDA) Adverse Event Reporting System (FAERS), among which over 17,000 cases were identified to have increased possibility of thyroid hyperplasia and neoplasm when taking glucagon-like peptide-1 (GLP-1) receptor agonist (RA) monotherapy. The data were compared to the cases where the patients were taking sodium-glucose cotransporter-2 (SGLT-2) inhibitor monotherapy. Please see my suggestions and concerns below.*

Response: We thank the reviewer for their thoughtful and constructive comments. Please see each of the concerns addressed below:

#### Suggestions and Concerns

*1. The authors compared data between GLP-1 RA monotherapy and SGLT-2 inhibitor monotherapy. It would be great to have a little bit more introduction or description for the readers to understand why they compared the data with SGLT-2 inhibitor monotherapy? What is the function of SGLT-2 inhibitors during the therapy? I realized that the authors described this a bit in the Methods section, but more information would be appreciated to be added in the Introduction section for the readers to understand the rationale.*

Response: Thank you for the suggestion. The *Introduction* section has been expanded with more details on SGLT-2 inhibitor’s mechanism of action and its place in diabetes management guidelines.

*2. In Table 1, how are the unique individual thyroid hyperplasia and/or thyroid neoplasm–related adverse event (AE) reports being counted? Was it the sum of the cases number searched by the above AE Preferred Term in each section, or it was counted through searching a specific AE Preferred Term?*

Response: Since there were cases in FAERS where multiple AEs occurred in the same report, we made sure that there was no overcount of the AEs by counting the unique cases rather than the total number of AE Preferred Terms. Table 1’s caption was expanded to clarify the definition.

*3. In the reporting odds ratio (ROR) analysis, where ROR = (a/b) / (c/d), I assume a indicates the number of AE cases in the exposed group, b indicates the number of non-AE cases in the exposed group, c indicates the number of AE cases in the control group, and d indicates the number of non-AE cases in control group. Is this correct? For calculating the ROR for all GLP-1 RAs (n=17,653; number of AEs=191) compared to the SGLT-2 inhibitor (n=14,102; number of AEs=7), maybe I am wrong, but should the ROR = (191 / [17,653−191 ]) / (7 / [14,102 − 7]), where (17,653 − 191) and (14,102 − 7) are the non-AE cases, which equals to 22.02? Did the authors use all the cases instead of the non-AE cases for the calculations? The same applies to the other ROR numbers and 95% CIs.*

Response: Yes, that is correct. Relative risk was calculated in error, instead of odds ratios. Thank you for catching the error! The numbers have been corrected in text and in the figure.

*4. The authors claimed that GLP-1 RA monotherapy reports manifested a statistically significant increase in thyroid hyperplasia and neoplasm AEs when compared to SGLT-2 inhibitors. How was the statistical significance determined? Was it because the calculated ROR is over 1 (or 10) or the interval (ROR [−], ROR [+]) is large?*

Response: Significance was determined by the ROR and the lower bound of the 95% CI being above 1.

*5. Figure 1’s resolution seems low in the document.*

Response: Thank you for the suggestion. A higher-resolution file has been uploaded to the journal.

## Abbreviations

AE: adverse event
FAERS: Food and Drug Administration Adverse Event Reporting System
FDA: Food and Drug Administration
GLP-1: glucagon-like peptide-1
RA: receptor agonist
ROR: reporting odds ratio
SGLT-2: sodium-glucose cotransporter-2
